# Combination of Elacridar with Imatinib Modulates Resistance Associated with Drug Efflux Transporters in Chronic Myeloid Leukemia

**DOI:** 10.3390/biomedicines10051158

**Published:** 2022-05-17

**Authors:** Raquel Alves, Ana Cristina Gonçalves, Joana Jorge, António M. Almeida, Ana Bela Sarmento-Ribeiro

**Affiliations:** 1 Laboratory of Oncobiology and Hematology (LOH) and University Clinic of Hematology, Faculty of Medicine University of Coimbra (FMUC), University of Coimbra, 3000-548 Coimbra, Portugal; raquel.alves@fmed.uc.pt (R.A.); jjorge@fmed.uc.pt (J.J.); 2 Coimbra Institute for Clinical and Biomedical Research (iCBR)—Group of Environmental Genetics of Oncobiology (CIMAGO), FMUC, University of Coimbra, 3000-548 Coimbra, Portugal; 3 Center for Innovative Biomedicine and Biotechnology (CIBB), 3004-504 Coimbra, Portugal; 4 Hospital da Luz Lisboa and Faculdade de Medicina, Universidade Católica Portuguesa, 1649-023 Lisbon, Portugal; amalmeida@ucp.pt; 5 CIIS (Centro de Investigação Interdisciplinar em Saúde), Universidade Católica Portuguesa de Lisboa, 1649-023 Lisbon, Portugal; 6 Hematology Service, Centro Hospitalar e Universitário de Coimbra (CHUC), 3000-061 Coimbra, Portugal

**Keywords:** imatinib resistance, ABC transporter inhibitors, elacridar, apoptosis

## Abstract

Multidrug resistance (MDR) development has emerged as a complication that compromises the success of several chemotherapeutic agents. In chronic myeloid leukemia (CML), imatinib resistance has been associated with changes in *BCR-ABL1* and intracellular drug concentration, controlled by SLC and ABC transporters. We evaluate the therapeutic potential of a P-glycoprotein and BCRP inhibitor, elacridar, in sensitive (K562 and LAMA-84) and imatinib-resistant (K562-RC and K562-RD) CML cell lines as monotherapy and combined with imatinib. Cell viability was analyzed by resazurin assay. Drug transporter activity, cell death, cell proliferation rate, and cell cycle distribution were analyzed by flow cytometry. Both resistant models presented an increased activity of BCRP and P-gP compared to K562 cells. Elacridar as monotherapy did not reach IC_50_ in any CML models but activated apoptosis without cytostatic effect. Nevertheless, the association of elacridar (250 nM) with imatinib overcomes resistance, re-sensitizing K562-RC and K562-RD cells with five and ten times lower imatinib concentrations, respectively. Drug combination induced apoptosis with increased cleaved-caspases-3, cleaved-PARP and DNA damage, reduced cell proliferation rate, and arrested CML cells in the S phase. These data suggest that elacridar combined with imatinib might represent a new therapeutic option for overcoming TKI resistance involving efflux transporters.

## 1. Introduction

The multidrug resistance (MDR) phenotype is a present challenge in multiple neoplasia management. Cancer cells become unresponsive to a large number of anticancer drugs, compromising the therapeutic response [1,2]. Several cellular/molecular mechanisms mediate this complex process, and one of the most exploited is the enhancement of drug efflux transporters that are responsible for reducing the intracellular drug concentration [2,3,4,5].

Chronic myeloid leukemia (CML) is a clonal hematological stem cell neoplasia resulting from a reciprocal translocation between chromosomes 9 and 22, that originating the *BCR-ABL1* fusion gene [6]. In this disease, the treatment is based on tyrosine kinase inhibitors (TKI) that block the action of the oncoprotein BCR-ABL [7]. Regardless of the excellent clinical results of TKIs, such as imatinib, the appearance of resistant cases is raised as a problem in clinical practice [8]. Alterations on the drug target, namely *BCR-ABL1* mutations, followed by factors that affect drug pharmacokinetics, are the mechanisms most associated with TKI resistance [9]. Particularly for non-receptor TKIs, such as imatinib, the action of drug efflux pumps is crucial to reaching a therapeutic concentration inside the target cells. Imatinib and other TKIs used in CML treatment are recognized as P-glycoprotein and BCRP substrates [10,11].

The contribution of efflux transporters from the ABC family to the MDR phenotype and the correlation of its expression with an inadequate response and lower survival rates led to the exploration of these pumps as therapeutic targets [12,13,14,15]. The inhibition of these efflux pumps will increase the concentrations of chemotherapeutic agents inside the cell, maximizing its effect [16,17]. Many molecules have been used as ABC transporter inhibitors to overcome drug resistance, with different results and specificity [18]. Also known as chemosensitizers, ABC inhibitors have been associated with secondary effects and toxicities, as described for verapamil and valspodar, making their clinical application challenging [18]. To minimize possible toxic effects of ABC inhibitors, several authors have studied the delivery of these inhibitors in nanoparticles in combination with other drugs [19,20]. Elacridar is a 3rd generation inhibitor developed to recognize not only P-gP, but also BCRP, as a target. This dual activity increases the interest for elacridar, making the inhibition of cancer stem cells possible, which are known to have an increased expression of P-gP and BCRP and be intrinsically resistant to therapies [21]. In multiple neoplasia models, the combined treatment of elacridar with anticancer agents re-sensitizes resistant cells [18,22,23,24]. In the phase I study, the combination of elacridar with topotecan in cancer patients resulted in substantial improvement in the bioavailability of topotecan with a safe elacridar toxicity profile [25]. The same safety profile was observed in the phase I trial in combination with doxorubicin [26]. However, in hematopoietic neoplasias, no studies have been carried out using elacridar, namely in imatinib resistance, contributing to the lack of clinical application of this ABC inhibitor.

In this study, we investigated the effects of elacridar as a single chemotherapeutic agent and in combination with imatinib, in sensitive and imatinib-resistant CML cell lines. We highlight the efficacy of lower doses of drugs in combination to reverse the imatinib resistance in models where the MDR phenotype is mediated by P-gP and BCRP activity.

## 2. Materials and Methods

### 2.1. Cell Lines and Cell Culture Conditions

We used four cell lines as CML models: K562 and LAMA-84 cells sensitive to imatinib and two imatinib-resistant cell lines—K562-RC and K562-RD cells. K562 and LAMA-84 cell lines were purchased from the American Type Culture Collection (ATCC), and the imatinib-resistant cells were developed in our laboratory based on continuous (K562-RC) and discontinuous exposure (K562-RD) to imatinib (Selleck Chemicals, Houston, TX, USA), as described by Alves et al. [27]. All the cells were maintained in an RPMI-1640 medium supplemented with 10% of FBS, 2 mM of L-glutamine, 100 U/mL of penicillin, and 100 μg/mL of streptomycin (GE Healthcare, Chicago, IL, USA) at 37 °C in a humidified atmosphere containing 5% CO_2_. According to the scheme of resistance, 250 nM of imatinib was added to the medium of resistant cell lines. The sensitive models presented the same *BCR-ABL1* transcript, however, in LAMA-84 cells, a Ph chromosome amplification was also described [28]. The IC_50_ of imatinib was 75 nM for K562, 140 nM for LAMA-84, 605 nM for K562-RC, and 1390 nM for K56-RD cells.

### 2.2. Drug Transporters Activity

The activity of P-gP and BCRP was detected in the four cell lines using the eFluxx-ID^®^ Green Multi-Drug Resistance Assay kit (ENZO, New York, NY, USA), according to manufacturer’s protocol. Briefly, cells were incubated with or without the MDR inhibitors (verapamil for P-gP and novobiocin for BCRP) in the presence of an eFluxx-ID probe for 30 min at 37 °C. To remove the dead cells from the analysis, propidium iodide (PI) solution (Biolegend, San Diego, CA, USA) was added during the last 5 min of incubation. After that, the cells were analyzed by flow cytometry (FC). The multidrug resistance activity factor (MRAF) was calculated based on the following equations using the mean fluorescence intensity (MFI) of the probe in each condition.

$${\mathrm{MRAF}}_{P-\mathrm{gP}}=\frac{{\mathrm{MFI}}_{\mathrm{verapamil}}-{\mathrm{MFI}}_{\mathrm{untreated}\;\mathrm{cells}}}{{\mathrm{MFI}}_{\mathrm{verapamil}}}\times 100$$

$${\mathrm{MRAF}}_{\mathrm{BCRP}}=\frac{{\mathrm{MFI}}_{\mathrm{novobiocin}}-{\mathrm{MFI}}_{\mathrm{untreated}\;\mathrm{cells}}}{{\mathrm{MFI}}_{\mathrm{novobiocin}}}\times 100$$

Results were expressed as a mean ± standard error of the mean (SEM) of five independent experiments.

### 2.3. Viability Assay

The resazurin assay was used to determine cell viability in the absence and presence of increasing concentrations of elacridar (Sigma-Aldrich, St. Louis, MO, USA) and/or imatinib in a single administration at 0 h. Both inhibitors were diluted in DMSO, and we used different stock solutions in order to add the same amount of solvent in each condition tested. For the combination studies, the cells were incubated with elacridar 0.25 μM plus increasing concentrations of imatinib. Briefly, the cells were plated at 0.5 × 10^6^ cells/mL and, after the treatment, resazurin was added to the cells, to a final concentration of 10 μg/mL for 2 h of incubation. The absorbance was measured at 570 nm and 600 nm, and the viability was calculated as a percentage of the control. The results were expressed as a mean ± SEM of nine independent experiments. 

### 2.4. Cell Death Analysis

Cell death was assessed through double staining with annexin V (AV) and 7-AAD by FC and by morphological evaluation using optical microscopy. After 48 h of incubation in the presence of elacridar, imatinib, or a combination of both drugs, the cells were washed with PBS by centrifugation at 400× *g* for 5 min. After that, the cells were stained with AV and 7-AAD (Biolegend, San Diego, CA, USA) and analyzed as described by Alves et al. [29]. Briefly, 0.5 × 10^6^ cells were collected and washed with PBS, centrifuged at 400× *g* for 5 min, resuspended in 100 μL of binding buffer and incubated with 5 μL of AV and 2 μL of 7-AAD for 15 min in the dark at room temperature. At least 25,000 events were acquired using CellQuest software (Becton Dickinson, Franklin Lakes, NJ, USA) and analyzed using Paint-a-Gate (Becton Dickinson, Franklin Lakes, NJ, USA). Results represent the percentage of each cell population: viable (AV^−^/7-AAD^−^), early apoptotic (AV^+^/7-AAD^−^), late apoptotic/ (AV^+^/7-AAD^+^), and non-apoptotic cell death (AV^−^/7-AAD^+^); and represent the mean ± SEM of six independents experiments. For morphological studies, the cells were seeded on glass slides and then stained with the May-Gründwald–Giemsa protocol [30]. Briefly, 1 × 10^6^ of untreated and treated cells were collected and seeded on glass slides. Then, smears were stained for 3 min with May-Grünwald solution (Sigma-Aldrich, St. Louis, MO, USA) and then for 15 min with Giemsa solution (Sigma-Aldrich, St. Louis, MO, USA). Cell morphology was analyzed by light microscopy using a Nikon Eclipse 80i microscope equipped with a Nikon Digital Camera DXM 1200 F.

### 2.5. Apoptosis, DNA Damage, and Cell Proliferation Analysis

The analysis of molecular markers of apoptosis, DNA damage, and cell proliferation was performed using the Apoptosis, DNA damage, and Cell proliferation kit (BD Pharmingen, Franklin Lakes, NJ, USA) plus anti-activated caspase 3 (BD Pharmingen, Franklin Lakes, NJ, USA), according to manufacturer’s instructions. After 48 h of treatment with different drugs, the cells were labeled with BrdU for 30 min at 37 °C. Then cells were washed, fixated, and permeabilized using BD Citofix Perm solution. Cells were stained with anti-BrdU (PerCP-Cy5.5), anti-cleaved PARP (PE), anti-H2AX (Alexa Fluor 647) and anti-activated caspase 3 (FITC), and analyzed by FC in a FACSCalibur cytometer (Becton Dickinson, Franklin Lakes, NJ, USA). Results were expressed as a percentage of positive cells for each molecular marker and represent the mean ± SEM of four independent experiments.

### 2.6. Cell Cycle Analysis

Cell cycle evaluation was performed in cells after 48 h of exposure to inhibitors, using PI solution with RNAse according to the manufacturer’s instructions. Briefly, 1 × 10^6^ of untreated and treated cells were collected and washed with PBS for 5 min at 400× *g*. The pellet was resuspended in 200 µL of 70% ethanol solution, during vortex agitation, and incubated for 30 min at 4 °C. Then, cells were washed with PBS, resuspended in 500 µL of PI/RNase solution (Immunostep, Salamanca, Spain) and finally analyzed by FC. ModFit LT software (Verity Software House, Topsham, ME, USA) was used to analyze the cell cycle distribution, and the results were expressed as the percentage of cells in each cycle phase (G_0_/G_1_, S, G_2_/M). Results represent the mean ± SEM of six independent experiments.

### 2.7. Statistical Analysis

Statistical analysis was performed with GraphPad Prism software, version 7.00 for Windows (GraphPad Software, San Diego, CA, USA). All values were expressed as the mean ± SEM of indicated independent experiences. The IC_50_ determination was performed by non-linear curve fit dose–response. Drug combination responses were calculated based on the highest single agent (HSA) model using SynergyFinder [31]. A HAS synergy score value >10 was considered synergistic, between −10 and +10 was considered additive, and a synergy score <−10 was considered antagonistic. A normality test was performed with a Kolmogorov–Smirnov test, and adequate analysis was used in accordance. A one-way ANOVA, Kruskal–Wallis test, Tukey’s test, and Dunnett’s and Dunn’s post hoc tests were used to determine statistical significance. In this study a *p*-value adjusted for the multiple comparison analysis of <0.05 was considered significant. Tukey’s test was used for comparison between the combination scheme and individual doses of each compound, and the Dunn’s or Dunnett’s test was used for comparison with untreated cells.

## 3. Results

### 3.1. Resistant Cell Lines Showed MDR Phenotype Mediated by P-gP and BCRP Activity

**Figure 1:**
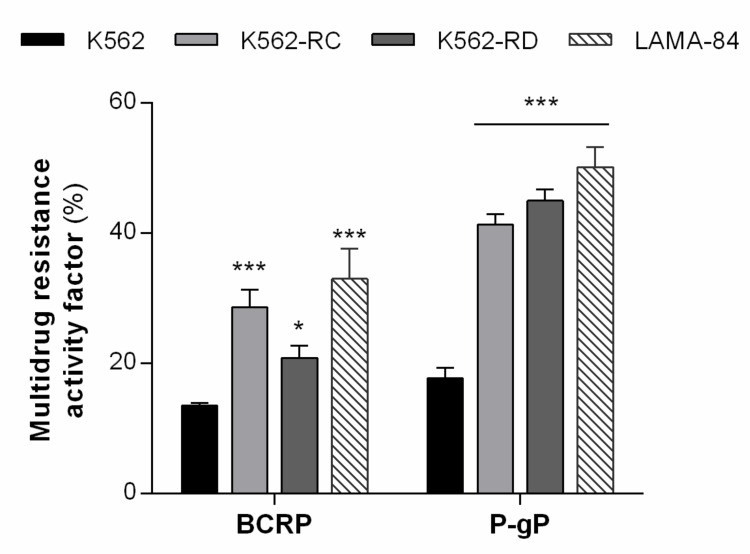
ABC transporters activity in CML models. The multidrug resistance activity factor (MRAF) represented the BCRP and P-gP activity in our cell lines and was determined using the eFluxx-ID^®^ Green Multi-Drug Resistance Assay kit. Results are expressed as a percentage (%) and represent the mean ± SEM obtained from five independent experiments. * *p* < 0.05; *** *p* < 0.001 (comparison with sensitive K562 cells).

In previous work, we observed that these models presented a significant increase in the percentage of cells expressing both drug transporters [27]. Since elacridar directly targets both P-gP and BCRP, we first investigated the activity of these ABC transporters on our cell lines (Figure 1). The resistant cells showed an increase in MRAF compared to K562-sensitive cells. The most altered transporter was P-gP in both models with over two times more activity compared with K562 cells (K562 17.7% vs. K562-RC 41.3% and K562-RD 45.0%, *p* < 0.001). Additionally, between the two sensitive cell lines, LAMA-84 showed over two times higher activity of both drug transporters compared with K562 cells (BCRP: K562 13.5% vs. LAMA-84 33.0%, *p* < 0.001; P-gP: K562 17.7% vs. LAMA-84 50.2%, *p* < 0.001). 

### 3.2. Combined Treatment with Elacridar Overcomes Imatinib Resistance

**Figure 2:**
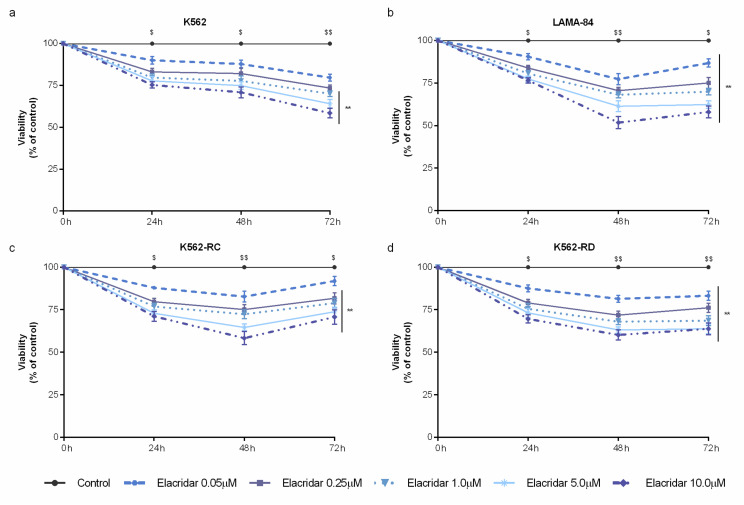
Dose–response curves of elacridar on sensitive and imatinib-resistant CML cell lines. K562 (**a**), LAMA-84 (**b**), K562-RC (**c**), and K562-RD (**d**) cells were incubated in the absence and presence of different concentrations of elacridar as monotherapy during 72 h. Results were expressed as a percentage (%) normalized to control and represent the mean ± SEM obtained from nine independent experiments. ** *p* < 0.01; comparison with control); ^$^ *p* < 0.05, ^$$^ *p* < 0.01 (comparison with time 0 h).

As a single agent, elacridar was able to decrease cell viability in a time– and dose–dependent manner (Figure 2). Despite the wide range of concentrations tested (from 0.05 μM to 10 μM), this inhibitor did not reach the IC_50_ in any cell line. Among the used cell lines, LAMA-84 cells were the most sensitive model to elacridar (Figure 2b), followed by the imatinib-resistant cells (Figure 2c,d), while K562 cells were the less sensitive cells to this ABC transporter inhibitor (Figure 2a). The mathematical IC_50_ was 39 μM for LAMA-84, approximately 80 μM for K562-RC, around 94 μM for K562-RD, and over 2000 μM for K562 cells after 48 h of treatment.

**Figure 3:**
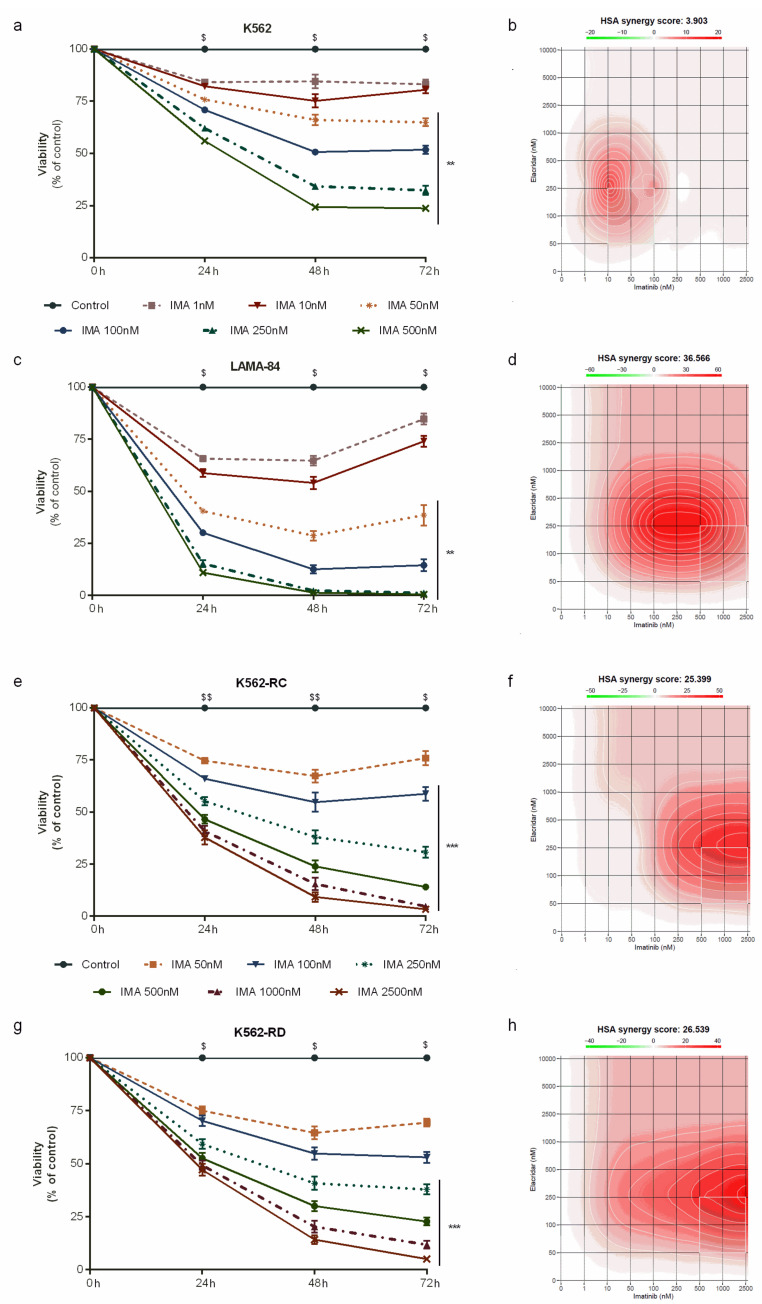
Dose–response curves of imatinib plus elacridar on sensitive and imatinib-resistant CML cell lines. K562 (**a**), LAMA-84 (**c**), K562-RC (**e**), and K562-RD (**g**) cells were incubated in the absence and presence of different concentrations of imatinib in combination with elacridar 0.25 μM during 72 h. The control cells correspond to cells treated with 0.25 μM of elacridar. Results were expressed as a percentage (%) normalized to control and represent the mean ± SEM obtained from nine independent experiments. The HSA synergy scores for imatinib plus elacridar were calculated using SynergyFinder 2.0 for K562 (**b**), LAMA-84 (**d**), K562-RC (**f**), and K562-RD (**h**) cells. A HAS synergy score value >10 was considered synergistic, between −10 and +10 was considered additive, and a synergy score <−10 was considered antagonistic. ** *p* < 0.01; *** *p* < 0.001 (comparison with control); ^$^ *p* < 0.05, ^$$^ *p* < 0.01 (comparison with 0 h). Ima: imatinib.

Due to this dual-targeting on P-gP and BCRP, we used a low dose of elacridar (0.25 μM) to overcome and improve the imatinib effect in our models. As shown in Figure 3, elacridar plus imatinib induced a decrease in cell viability in time–, dose– and a cell-line–dependent manner. 

In the sensitive cell line, the combination of both drugs increased the IC_50_ of imatinib from 75 nM to 92 nM in K562 cells (Figure 3a), while in LAMA-84 the IC_50_ of imatinib decreased over 20 times, from 140 nM to 6.2 nM (Figure 3c). In contrast to parental cells, in resistant cells we observed an increase in imatinib sensitivity when associated with elacridar. For K562-RC cells, the IC_50_ of imatinib reduced from 605 nM to 126 nM, almost five times lower (Figure 3e). The discontinuous model presented an IC_50_ of imatinib of 1390 nM that decreased to 133 nM in combination with elacridar, over ten times lower (Figure 3g). The combination of both inhibitors reduced the degree of resistance from 8.0 and 18.4 times to 1.7 and 1.8 for K562-RC and K562-RD, respectively. Additionally, we determined the synergy score for the imatinib and elacridar combination with SynergyFinder, using the HSA model. For the K562 cells, the HAS synergy score was 3.9 (Figure 3b), while in imatinib-resistant cells, this synergy score was higher than 25, confirming a strong synergism (25.4 for K562-RC and 26.5 for K562-RC; Figure 3f,h). The highest synergy score (36.6) was observed in LAMA-84 cells. In further studies, we focus on imatinib-resistant cells and the parental sensitive cells (K562).

### 3.3. Apoptosis as the Main Mechanism of Cell Death in the Combined Treatment

**Figure 4:**
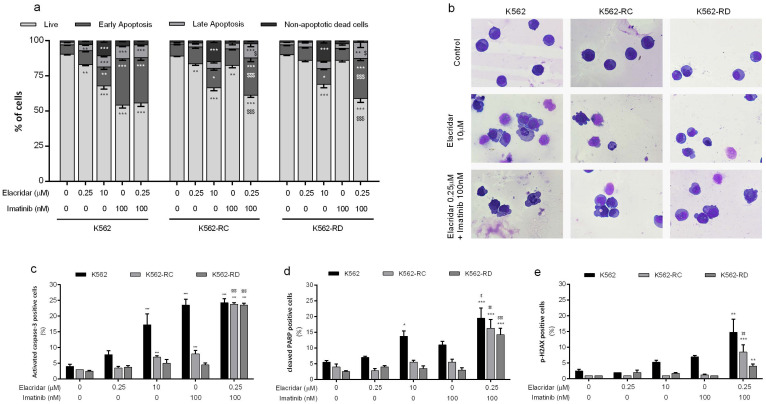
Analysis of cell death induced by elacridar as monotherapy and in combination with imatinib in CML cell lines. (**a**) The type of cell death was identified by annexin V/7-AAD staining and analyzed by flow cytometry; data were expressed as a percentage (%) of live, early apoptotic, late apoptotic/, and non-apoptotic cell death. In (**b**), cell morphology was analyzed in smears stained with May-Grünwald–Giemsa (amplification: 500×). The expression levels of activated caspase-3 (**c**), cleavage PARP (**d**), and phosphorylated-H2AX (**e**) were determined by flow cytometry as markers of apoptosis and DNA damage. Results were obtained after 48 h of incubation and represent mean ± SEM of at least four independent experiments. * *p* < 0.05; ** *p* < 0.01; *** *p* < 0.001 (comparison with control); ^$^ *p* < 0.05, ^$$^ *p* < 0.01; ^$$$^ *p* < 0.001 (comparison with lower dose of correspondent inhibitor).

We evaluated the mechanism of cell death induced by elacridar as monotherapy for the doses of 0.25 μM and 10 μM, using AV and 7-AAD (Figure 4a). The highest dose of elacridar induced cell death by apoptosis, confirmed by the increase in cells in early apoptosis and late apoptosis (*p* < 0.001 compared to control cells). The apoptosis initiation was supported by the significant increase in activated caspase-3-positive cells and the increase in cells with cleaved PARP (a marker of apoptosis) (Figure 4c,d). In addition to apoptosis, this inhibitor activated other mechanisms of cell death, supported by the increase in non-apoptotic dead cells (*p* < 0.001 compared to control cells). In the same line, the morphological analysis showed the presence of apoptotic cells, characterized by blebbing of the cell membrane and nucleus fragmentation, as well as by the presence of non-apoptotic dead cells identified by the release of the cytoplasmatic content due to membrane destabilization and an unaltered nucleus (Figure 4b).

The combined treatment of elacridar plus imatinib induced a significant decrease in viable cells with an increase on the early apoptosis population. In resistant models, these differences were not only statistically significant (*p* < 0.001) when compared with untreated cells, but also when compared with both isolated inhibitors (*p* < 0.001). All cell lines showed a significant increase in the percentage of positive cells for apoptosis markers (*p* < 0.01) and an increase in the percentage of cells positive for p-H2AX (a marker for DNA damage, *p* < 0.01) (Figure 4e) when treated with elacridar plus imatinib. In accordance with other results, the morphological analysis of this condition showed alterations typical of apoptotic cell death (Figure 4b).

### 3.4. Drug Combination Reduces Cell Proliferation and Alters Cell Cycle Distribution

**Figure 5:**
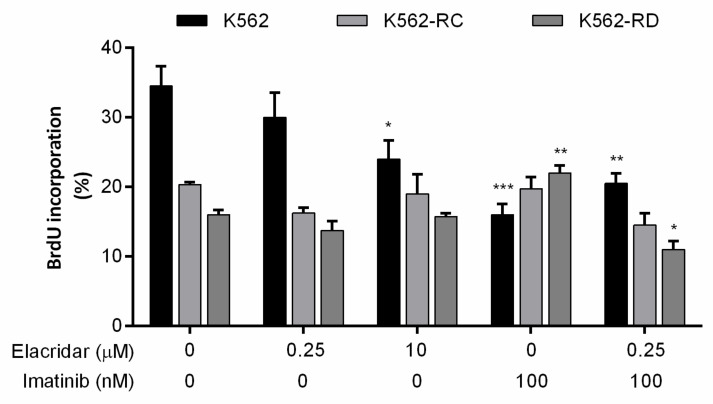
Effect of elacridar as monotherapy and in combination with imatinib on cell proliferation of CML cell lines. BrdU incorporation was evaluated to determine the cell proliferation rate of CML cell lines after each treatment, using Apoptosis, DNA damage, and Cell Proliferation kit for flow cytometry. Results were obtained after 48 h of incubation and represent mean ± SEM of at least four independent experiments. * *p* < 0.05; ** *p* < 0.01; *** *p* < 0.001 (comparison with control).

The incorporation of BrdU works as a marker for cell proliferation, and we evaluated this marker in the different cell lines according to each treatment (Figure 5). Untreated cells point out the different profiles of proliferation of each model, where K562 cells were more proliferative than resistant cell lines. The opposite was observed with imatinib 100 nM treatment, where resistant cell lines proliferate more in the presence of TKI, while a significant decrease was observed in sensitive cells, proving the resistant phenotype. Elacridar as monotherapy only reduced the proliferation rate of K562 with the highest dose, although no alterations were observed in resistant models. However, in the combined therapy of elacridar plus imatinib, a reduction was observed in the cell proliferation rate in all models (Figure 5).

The cytostatic effect of these inhibitors was also evaluated by cell cycle distribution (Table 1). In K562 cells, the dose of 10 μM of elacridar decreased the S phase and induced slightly increased cells in the G_2_/M phase (Table 1). In the same cell line, drug combination treatment induced an increase in cells in the G_0_/G_1_ phase with a decrease in cells in the S phase, revealing a cytostatic effect on these cells. The changes were statistically significant when compared with both drugs as monotherapy. In resistant models (Table 1), corroborating the results of BrdU incorporation, we observed an increase in cells in the S phase when exposed to imatinib as monotherapy. The distribution of cells through the cell cycle phases in K562-RC and K562-RD cells were very similar between imatinib as monotherapy and the combination of both drugs, with a reduction in cells in G_0_/G_1_ and an increase in the S phase.

## 4. Discussion

The crucial role of ABC transporters on TKI accumulation and their activity as a mechanism of resistance in CML led to exploiting some ABC transporter inhibitors to overcome TKI resistance. In this study, we evaluated the therapeutic potential of elacridar, a dual P-gP and BCRP inhibitor, in CML in vitro models. This study demonstrated that resistant models were more sensitive to elacridar than the parental sensitive cells (K562). Even among imatinib-sensitive models, we observed a more pronounced effect in LAMA-84, that presents the highest P-gp and BCRP activity. However, as a single chemotherapeutic agent, this inhibitor did not reveal promising results. The induction of cell death was mediated by apoptosis but this was only achieved with higher doses (10 μM). Nevertheless, the combination of lower doses of elacridar may be sufficient to block imatinib extrusion from leukemic cells, improving BCR-ABL inhibition by the TKI. To analyze this hypothesis, in our study, we combined elacridar at a lower dose (0.25 μM) with imatinib to attempt an improved response or reverse TKI resistance. This combination showed high efficacy and significantly reduced the degree of resistance of our models, and led to a better response with low drug concentration in sensitive cells that present significant drug transporter activity (LAMA-84 cells). The overcoming of TKI resistance was promoted by activation apoptosis and a decrease in cell proliferation rate.

P-gP has been extensively studied and its activity was associated with low response rates, early recurrence and poor survival in different types of cancer [16,22,32]. These characteristics made this transporter an attractive drug target. In the blood–brain barrier, the activation of BCRP-mediated transport has been described as a compensatory mechanism in the case of P-gP inhibition or loss of expression [33]. BCRP expression has been frequently described in several neoplasias, like breast cancer and hematological malignancies [12], and particularly associated with cancer stem cells [21,34,35]. Furthermore, in most cancers, relapse or recurrence has an origin in cancer stem cells that seems to be insensitive to therapy [36]. In CML, a clonal hematopoietic stem cell disorder, the acquisition of TKI resistance can have multiple roots where both P-gP and BCRP have a crucial role in limiting TKI intracellular concentration [37]. Additionally, some results point out the higher affinity of imatinib to BCRP rather than to P-gP [38]. Supporting the contribution of both ABC transporters to resistance, our imatinib-resistant models show increased activity of both pumps. Taking this into consideration, we selected the third generation P-gP inhibitors for our study—elacridar. This dual P-gP and BCRP inhibitor was specifically designed to the treatment of MDR cancers, avoiding CYP450 interaction [39]. It is an oral inhibitor that acts through ATP hydrolysis inhibition and consequently blocks the transport activity [18].

As described by Planting et al., elacridar by itself does not present antitumor activity [26], corroborating our results obtained as monotherapy. However, this inhibitor showed great potential as a chemosensitizer for combined treatment with minimal side effects at doses needed for transporter inhibition [18,25,26]. Additionally, the combination with lower doses of elacridar may also promote a higher oral availability and distribution of antitumor agents in tissues of difficult access, such as the brain [40,41,42]. According to Bruin et al., the dose of elacridar needed to inhibit P-gP and BCRP were different, being 0.05–0.1 μM and 0.25 μM, respectively [43]. Based on this data, in our work we used the dose of 0.25 μM of elacridar in combination treatment with imatinib to guarantee the block of both transporters. In agreement with our results, the use of this drug overcomes the resistance to other cytotoxic drugs, such as irinotecan and paclitaxel, in cancer-resistant models [44,45]. In lung cancer cell lines, the combination of 0.25 μg/mL (≈0.444 μM) of elacridar with docetaxel was able to restore drug sensitivity [23]. The same synergistic effect was observed with bortezomib in a multiple myeloma cell line, RPMI-DOX40, and with sunitinib in renal carcinoma cell lines [22,24]. In mouse models, elacridar potentiated in the 60-fold activity of doxorubicin in P338/Dox-resistant leukemia cells [46]. Moreover, some authors reported the reduction in IC_50_ of some chemotherapeutic agents, not only in resistant models, but also in sensitive cells [44,45]. In our results, the combined scheme was not useful in K562 cells, probably due to low transporters expression, while in LAMA_84, sensitive models with high drug transporter activity led to a 20 times reduction in the imatinib IC_50_. In some cases, a combination of several drugs did not present a complete reversal of resistance, suggesting the involvement of other cellular mechanisms and demonstrating the complexity of drug resistance [3]. In our models, the reversal of the resistance degree was almost complete, reducing from 8.0 and 18.4 times to 1.7 and 1.8 in K562-RC and K562-RD, respectively. These results could be justified by another resistance mechanism previously identified in these models, such as low expression of influx proteins and *BCR-ABL* overexpression [27]. 

The broad expression of ABC transporters through the body makes the translation of in vitro and in vivo results challenging in clinical settings [14]. The use of this type of inhibitor could affect the pharmacokinetics of co-administered drugs, leading to undesirable toxic effects, as observed in some clinical trials [14,47]. With old generation MDR reversal agents, one of the problems was the interaction with metabolism enzymes [47]. However, elacridar was designed to prevent this type of interaction; only with doses over 10 μM is it able to affect, for instance, CYP3A4 [39]. To prevent this interaction and to avoid adverse effects, the combination scheme should be based on lower doses of both inhibitors. Another aspect that should be taken into consideration is the impact of elacridar or other P-gP inhibitors on excretion, since this interaction could promote an increase in the systemic circulation of drugs [18]. From our results and other works, low doses of elacridar should be sufficient to promote a synergic effect with other cytotoxic drugs and, if possible, match the pharmacokinetics of both drugs to maximize results [18]. Although not yet in clinical practice, the characteristics of elacridar and the results obtained in different models made this molecule an attractive new therapeutic agent, particularly to circumvent the MDR phenotype. 

## 5. Conclusions

Our results highlight the contribution of ABC transporter activity to the CML-resistance phenotype and their possible role as therapeutic targets. The combination of a low dose of elacridar and imatinib seems an advantageous therapeutic option for cases of TKI-resistance that involves P-gP and BCRP activity.

## Figures and Tables

**Table 1:** Effects of elacridar in the cell cycle of imatinib-sensitive and imatinib-resistant cells.

		G_0_/G_1_ (%)	S (%)	G_2_/M (%)
K562 Cells				
	Control	39.7 ± 0.7	49.0 ± 0.6	11.8 ± 0.9
	Elacridar 0.25 μM	37.0 ± 1.4	51.3 ± 0.7	11.7 ± 1.7
	Elacridar 10 μM	41.2 ± 1.6	41.8 ± 1.6 **	17.0 ± 1.8
	IMA 100 ηM	38.3 ± 2.1	47.3 ± 0.8	14.3 ± 1.7
	Elacridar 0.25 μM + IMA 100 ηM	46.2 ± 2.7 ^$^	41.3 ± 2.4 **^,$^	12.5 ± 2.3
K562-RC Cells				
	Control	67.3 ± 2.2	22.5 ± 1.4	10.2 ± 0.8
	Elacridar 0.25 μM	65.2 ± 3.2	23.8 ± 2.2	11.0 ± 1.0
	Elacridar 10 μM	62.5 ± 3.4	23.5 ± 1.6	14.0 ± 1.9
	IMA 100 ηM	51.8 ± 2.2 **	38.2 ± 0.9 ***	10.0 ± 1.3
	Elacridar 0.25 μM + IMA 100 ηM	52.7 ± 1.9 **	37.5 ± 1.4 ***	9.8 ± 0.8
K562-RD Cells				
	Control	63.2 ± 1.2	26.8 ± 1.1	10.2 ± 0.3
	Elacridar 0.25 μM	60.0 ± 2.3	29.0 ± 2.1	11.0 ± 0.4
	Elacridar 10 μM	63.8 ± 2.4	23.3 ± 2.1	13.0 ± 0.4
	IMA 100 ηM	51.3 ± 2.1 ***	37.8 ± 1.9 ***	10.8 ± 0.4
	Elacridar 0.25 μM + IMA 100 ηM	54.5 ± 1.7 *	32.7 ± 1.2	12.8 ± 1.2

* *p* < 0.05, ** *p* < 0.01, *** *p* < 0.001 comparing with control; ^$^ *p* < 0.05, comparing with lower dose of correspondent inhibitor. IMA: imatinib.

## Data Availability

All data generated or analyzed during this study were included in this published article.
